# Lactococcus lactis subsp. cremoris Reprograms the Gut-Liver Axis and Protects Against Liver Injury

**DOI:** 10.64898/2026.09.16.752188

**Published:** 2026-09-18

**Authors:** Camilo Anthony Gallarde Gacasan, Jacyln Weinberg, Gabrielle M Webster, Crystal R Naudin, ViLinh Tran, Lauren C Askew, Maria E Barbian, Dean P Jones, Rheinallt M Jones

## Abstract

Metabolic diseases are increasingly linked to dysregulation of the gut-liver axis, highlighting the therapeutic potential of probiotics. Lactococcus lactis subsp. cremoris (LLC) protects against experimental hepatic steatosis, but the mechanisms underlying its benefits remain poorly understood. Here, we integrated untargeted metabolomics, gnotobiotic mouse models, targeted bile acid profiling, and liver injury paradigms to systematically define LLC-mediated metabolic reprogramming. LLC extensively remodeled serum and hepatic metabolomes in Western diet-fed mice, enriching pathways associated with lipid metabolism, xenobiotic biotransformation, and redox homeostasis. LLC attenuated ethanol-induced steatosis and acetaminophen-induced liver injury, accompanied by activation of Nrf2-dependent antioxidant programs and FXR signaling. LLC monocolonization was sufficient to reprogram the hepatic metabolome, revealing direct host metabolic effects. Reduced intestinal bile acid conjugation emerged as a prominent LLC-associated phenotype. Together, these findings identify LLC as a probiotic that reprograms the gut-liver metabolic axis to enhance metabolic resilience and hepatoprotection.

## Full Text Availability

The license terms selected by the author(s) for this preprint version do not permit archiving in PMC. The full text is available from the preprint server.

